# Oxytocin and socioemotional aging: Current knowledge and future trends

**DOI:** 10.3389/fnhum.2013.00487

**Published:** 2013-08-28

**Authors:** Natalie C. Ebner, Gabriela M. Maura, Kai MacDonald, Lars Westberg, Håkan Fischer

**Affiliations:** ^1^Department of Psychology, University of FloridaGainesville, FL, USA; ^2^Department of Psychiatry, University of CaliforniaSan Diego, La Jolla, CA, USA; ^3^Department of Pharmacology, University of GothenburgGothenburg, Sweden; ^4^Department of Psychology, Stockholm UniversityStockholm, Sweden; ^5^Aging Research Center, Karolinska InstituteStockholm, Sweden

**Keywords:** oxytocin, aging, socioemotional functioning, amygdala, anterior cingulate

## Abstract

The oxytocin (OT) system is involved in various aspects of social cognition and prosocial behavior. Specifically, OT has been examined in the context of social memory, emotion recognition, cooperation, trust, empathy, and bonding, and—though evidence is somewhat mixed-intranasal OT appears to benefit aspects of socioemotional functioning. However, most of the extant data on aging and OT is from animal research and human OT research has focused largely on young adults. As such, though we know that various socioemotional capacities change with age, we know little about whether age-related changes in the OT system may underlie age-related differences in socioemotional functioning. In this review, we take a genetic-neuro-behavioral approach and evaluate current evidence on age-related changes in the OT system as well as the putative effects of these alterations on age-related socioemotional functioning. Looking forward, we identify informational gaps and propose an *Age-Related Genetic, Neurobiological, Sociobehavioral Model of Oxytocin* (*AGeNeS-OT model*) which may structure and inform investigations into aging-related genetic, neural, and sociocognitive processes related to OT. As an exemplar of the use of the model, we report exploratory data suggesting differences in socioemotional processing associated with genetic variation in the oxytocin receptor gene (*OXTR*) in samples of young and older adults. Information gained from this arena has translational potential in depression, social stress, and anxiety-all of which have high relevance in aging—and may contribute to reducing social isolation and improving well-being of individuals across the lifespan.

Social and emotional processes and their associated genetic and neurobiological mechanisms in aging are still incompletely understood (Nielsen and Mather, 2011). In this paper we propose to combine neuroendocrine, genetic, and sociobehavioral approaches to examine the role of the oxytocin (OT) system in the context of socioemotional aging. Aspects of the OT system warranting investigation include: (1) changes in endogenous and dynamic OT levels; (2) changes in systems which directly impact OT function (i.e., gonadal hormones); (3) genetic variation in aspects of the OT system, including the gene for oxytocin (*OXT*), its receptor (*OXTR*), and the related CD38 system; (4) changes in OT-rich neural regions; (5) the effect of exogenous OT. There is increasing evidence that OT plays a significant role in many of the socioemotional capacities that undergo age-related changes. However, to date, very little is known about the role of OT in human aging (Huffmeijer et al., 2012). Thus, it will be crucial for future research to clarify links between age-related changes in the aforementioned aspects of the OT system and changes in neural processing and subsequent alterations in experience as well as behavior in socioemotional domains in older compared to young adults.

To foreshadow, this focused review conceptually integrates two lines of research. First, we summarize evidence for age-associated changes in socioemotional capacities (Isaacowitz et al., 2007; Ruffman et al., 2008; Scheibe and Carstensen, 2010). Second, we review evidence for the involvement of OT in socioemotional functioning (Bartz et al., 2011; Meyer-Lindenberg et al., 2011; Van IJzendoorn and Bakermans-Kranenburg, 2012). Synthesizing these two lines of work, we present an *Age-Related Genetic, Neurobiological, Sociobehavioral Model of Oxytocin* (*AGeNeS-OT model*) which may stimulate questions and organize investigations into the role of OT in socioemotional aging. As an example of the use of the *AGeNeS–OT* model, we report preliminary data suggesting neural and behavioral differences in socioemotional processing associated with genetic variations in *OXTR* in samples of young and older adults. We conclude by suggesting future directions for research implied by the model. Ultimately, these investigations will increase our understanding of the role of OT in aging and will have the potential for generating new interventions to improve health and well-being.

## Socioemotional functioning and aging

From life's beginning, humans are confronted with critical, survival-enhancing socioemotional stimuli related to self and others. To maintain successful social interactions and avoid the negative consequences of social isolation (Baumeister and Leary, 1995; Norman et al., 2011), we learn to quickly and accurately process, respond to, and remember social cues (Baron-Cohen et al., 2000; Grady and Keightley, 2002; Adolphs, 2003). Socioemotional functioning may become particularly relevant in old age when-due to the experience of increasing physical ailment, dependency, and age-related social losses-the experience of social isolation often increases with negative effects on physical and mental health (Cornwell and Waite, 2009).

The extant literature suggests a mixed picture of age-related changes in socioemotional capabilities: Some capacities (e.g., emotion regulation, emotional problem solving) improve with age, whereas other skills (e.g., recognition of emotions in others) decline (cf. Scheibe and Carstensen, 2010). In particular, across various studies, older compared to young adults show increased emotion regulation capacity (Carstensen, 2006; Blanchard-Fields et al., 2007; Riediger et al., 2009; Scheibe and Blanchard-Fields, 2009; Voelkle et al., 2013) and greater confidence in this ability (Lawton et al., 1992; Gross and Levenson, 1997; Kessler and Staudinger, 2009). The majority of older adults are well-adjusted emotionally and report relatively high levels of affective well-being and emotional stability as documented in cross-sectional (Carstensen et al., 2000) as well as longitudinal (Carstensen et al., 2011) studies (see also Charles et al., 2001; Teachman, 2006). In addition, older compared to young adults are at least equally (and often more) effective in their ability to regulate their emotional experiences, autonomic arousal, and outward display of negative emotions in language and faces when instructed to do so (Kunzmann et al., 2005; Magai et al., 2006; Phillips et al., 2008), and show improved socioemotional problem solving capacity (Blanchard-Fields et al., 2007).

**Figure 1:**
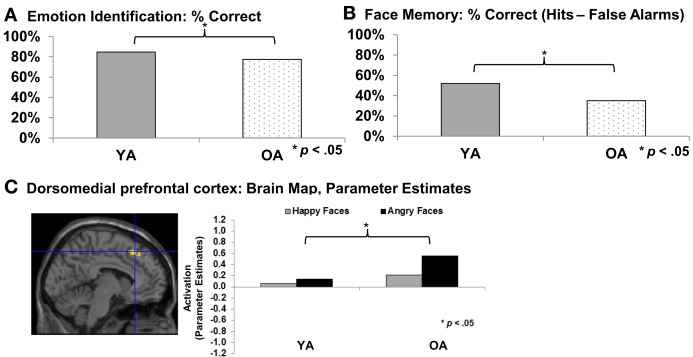
**(A)** Emotion identification (Ebner et al., 2010); **(B)** Face memory (Ebner and Johnson, 2009); **(C)** Emotion identification: dorsomedial prefrontal cortex (Ebner et al., 2012). YA, Young adults; OA, Older adults.

At the same time, older adults often show increased difficulties in accurate recognition of social and emotional cues (for reviews see Isaacowitz et al., 2007; Ruffman et al., 2008; see also Ebner and Johnson, 2010; see Figure 1A). Recent functional magnetic resonance imaging (fMRI) data suggests that these difficulties are associated with greater activity in dorsomedial prefrontal cortex (dmPFC) in older compared to young adults during facial emotion reading, particularly for angry expressions (Williams et al., 2006; Keightley et al., 2007; Ebner et al., 2012; see Figure 1C). This association comports with previous evidence that dmPFC is involved in complex processing and cognitive and emotional control (Amodio and Frith, 2006). Another age-related change in socioemotional functioning is that older compared to young adults demonstrate more interpersonal trust (List, 2004; Castle et al., 2012). This change may be due to the difficulty older adults often have in “reading” the emotions of others, as suggested by recent findings that older compared to young adults are less proficient at detecting lies, mediated by deficits in emotion recognition (Ruffman et al., 2012). With respect to changes in memory, there is evidence that the majority of older adults experience declines in remembering critical socioemotional cues, including names (Crook et al., 1993; Verhaeghen and Salthouse, 1997) and faces (Bartlett et al., 1989; Grady et al., 1995; Ebner and Johnson, 2009; see Figure 1B). Finally, in terms of social motivation, there is robust evidence that older adults are more avoidance-oriented and less approach-oriented than young adults (Ebner et al., 2006; Freund, 2006; Nikitin et al. in revision).

Importantly, the mechanisms underlying these age-related changes in socioemotional functioning are not well-understood yet. One potential explanation is differences in visual processing (Isaacowitz et al., 2006; Ebner et al., 2011), perhaps as a function of age-related changes in motivation (Mather and Carstensen, 2005; Carstensen, 2006; Samanez-Larkin and Carstensen, 2011). In particular, there is evidence that older compared to young adults spend more time looking at positive than negative information (Isaacowitz et al., 2006) and, when processing faces, spend less time viewing the eye region and more time viewing the mouth (Firestone et al., 2007). This age-differential visual processing pattern may be important given that the eye vs. mouth regions of a face carry different socioemotional information (Calder et al., 2000; Ebner et al., 2011).

A complementary, mechanistic explanation for age-related changes in socioemotional function may be changes in brain structure or function in regions associated with socioemotional processing such as amygdala, PFC, insula, or fusiform gyrus (Keightley et al., 2007; Grady, 2008; Cacioppo et al., 2009; Ebner et al., 2012; see Ruffman et al., 2008; Samanez-Larkin and Carstensen, 2011; St. Jaques et al., 2013, for overviews). For instance, there is well-documented, age-related structural decline in regions such as the lateral PFC (lPFC), insula, and striatum (Raz, 2005; Raz et al., 2005). Regarding functional changes, one common finding is an age-related decrease in amygdala activation during the perception of emotional stimuli (especially negative stimuli) accompanied by an age-related increase in activity in a number of lPFC and mPFC regions (Iidaka et al., 2002; Gunning-Dixon et al., 2003; Fischer et al., 2005, 2010; Tessitore et al., 2005; but see Mather and Carstensen, 2005; Wright et al., 2006; Ebner et al., 2013).

Crucially, however, extant literature suggests that age-related differences in socioemotional processing cannot be explained solely by age-related visuoperceptual and/or neurocognitive changes (Samanez-Larkin and Carstensen, 2011). In addition, it may be that changes in socioemotional function are also linked with age-related alterations in neuroendocrine function. In particular, the neuropeptide OT appears as a particularly promising candidate, given increasing evidence of its role in socioemotional domains (Insel and Fernald, 2004; Donaldson and Young, 2008; Bartz et al., 2011; Meyer-Lindenberg et al., 2011; Norman et al., 2011). However, to date, we know very little about age-related changes in the OT system, particularly in the context of socioemotional aging (Huffmeijer et al., 2012).

## Oxytocin and socioemotional functioning

OT is a nine amino acid peptide, with peripheral and central functions (Gimpl and Fahrenholz, 2001). It is synthesized in magnocellular neurosecretory cells of paraventricular nuclei (PVN) and supraoptic nuclei (SON) of the hypothalamus and released through the posterior pituitary gland into the periphery (Insel, 2010). OT is also released into the brain by magnocellular dendrites (Leng and Ludwig, 2006) and by OT-releasing neurons projecting to specific brain regions such as the amygdala, hippocampus, and striatum (Kimura et al., 1992; Landgraf and Neumann, 2004; Knobloch et al., 2012). Human and animal studies combined suggest that the function of the OT system is reflected at a variety of physiological and anatomical levels, including: (1) peripheral hormone levels (i.e., plasma and saliva); (2) central hormone levels [i.e., in cerebrospinal fluid (CSF)]; (3) histological levels (i.e., presence and size of OT cells); (4) receptor levels (in OT receptor binding in defined brain regions); (5) genetic levels, or the level of “neuropeptidergic individuality” (MacDonald, 2012); i.e., polymorphisms related to *OXT* or *OXTR*, or genes related to OT release (i.e., *CD38*; Sauer et al., 2012, 2013).

In particular, accumulating evidence suggests that OT may serve as a key effector in socioemotional functioning such as emotion recognition, memory for faces, interpersonal trust, and bonding as briefly summarized next (see Bartz et al., 2011; Meyer-Lindenberg et al., 2011; Norman et al., 2011; Zink and Meyer-Lindenberg, 2012, for comprehensive overviews).

After the discovery that certain neuropeptides could be delivered intranasally to the human brain (Born et al., 2002), a number of experimental studies using intranasal OT revealed intriguing effects on diverse aspects of socioemotional functioning. For example, research in healthy adults suggests that OT impairs performance in verbal memory tasks (Ferrier et al., 1980; Heinrichs et al., 2004; but see Feifel et al., 2012), while enhancing recognition of social (i.e., faces) but not non-social stimuli (Rimmele et al., 2009; see also Heinrichs et al., 2004), especially for neutral and angry compared to happy faces (Savaskan et al., 2008). Furthermore, intranasal administration of OT increases overall gaze time toward faces (Guastella et al., 2008; Andari et al., 2010; Averbeck, 2010; Gamer et al., 2010) and increases emotion recognition, specifically of happy and fearful faces (and under certain conditions angry faces; see Shahrestani et al., 2013, for a recent review).

In addition, recent studies have shown that intranasal OT increases facial trustworthiness and attractiveness ratings (Theodoridou et al., 2009) as well as interpersonal trust and the willingness to take social risks (Kosfeld et al., 2005; Baumgartner et al., 2008; Phan et al., 2010). These effects of OT on trust seem to be particularly pronounced in positive social interactions (Zak et al., 2005; Mikolajczak et al., 2010) and with respect to in-group vs. out-group members (Van IJzendoorn and Bakermans-Kranenburg, 2012). Moreover, these effects seem moderated by interindividual differences (Rockliff et al., 2011; but see Guastella et al., 2013), including genetic polymorphisms associated with OT function (Riedl and Javor, 2011; see also Rodrigues et al., 2009; MacDonald, 2012, for reviews).

Besides these effects on facial processing and trust, intranasal OT has been shown to influence social approach behavior, attachment, bonding, and social rejection with associated health benefits (Ditzen et al., 2009; Gouin et al., 2010; Scheele et al., 2012; Schneiderman et al., 2012; Fekete et al., 2013). For example, intranasal OT increased positive relative to negative behaviors during a laboratory couple conflict and reduced post-conflict cortisol levels (Ditzen et al., 2009). This potential stress reducing-effect of OT has been further documented by evidence that participants with increased plasma OT healed faster and had a greater number of positive interactions with partners during a 24-h hospital stay (Gouin et al., 2010; see also Kéri and Kiss, 2011; Kiss et al., 2011; see Taylor et al., 2006, for a discussion of OT's role during relaxation vs. stress; see also Feldman et al., 2011).

An ever-expanding body of neuroimaging data suggests that OT's effects on socioemotional functioning are due to its attenuation of the neural circuitry for anxiety and aversion and its activation of social reward neural networks (cf. Yoshida et al., 2009; Zink and Meyer-Lindenberg, 2012). In particular, a number of studies have provided evidence that the amygdala might be a key structure for the mediation of the social-cognitive effects of OT (Kirsch et al., 2005; Domes et al., 2007a; Petrovic et al., 2008; Singer et al., 2008; Labuschagne et al., 2010; Riem et al., 2011; Zink and Meyer-Lindenberg, 2012; cf. Huffmeijer et al., 2012; but see Domes et al., 2010). For example, OT attenuates amygdala response to fear-inducing stimuli (Kirsch et al., 2005). Baumgartner et al. (2008; see also Kosfeld et al., 2005; Mikolajczak et al., 2010) provide evidence that OT reduced betrayal aversion to breaches of trust via a reduction in bilateral amygdala activation and midbrain regions and greater ventral striatum and orbitofrontal cortex (OFC) activity. Furthermore, there are suggestions of specific modulatory influences of OT on subregions within the amygdala during processing of socioemotional information (Gamer et al., 2010; see also Huber et al., 2005; Viviani et al., 2011; Knobloch et al., 2012). These central effects, importantly, occur in interaction with a network of other neurochemicals including estrogen, dopamine, and serotonin (Riedl and Javor, 2011).

Thus there are suggestions in the literature that OT increases approach-related behaviors, while decreasing withdrawal-related behaviors (Kemp and Guastella, 2010). At the same time, however, there is evidence suggesting that OT may play a somewhat more complex role in social behavior than simply directing approach vs. avoidance behavior and/or attentional biases to positive and negative information, respectively. Rather, OT may increase social engagement, salience of social agents, and social value of processed information, largely independent of valence (Shamay-Tsoory et al., 2009; Tops, 2009; Shamay-Tsoory, 2010; Bartz et al., 2011). In line with this suggestion, brain regions such as the ventral tegmentum, PFC, nucleus accumbens, and insula associated with the social-reward neural network have shown sensitive to OT (Balleine et al., 2007; Riem et al., 2011; Wittfoth-Schardt et al., 2012; Groppe et al., 2013; Scheele et al., 2013).

The central effects of OT are mediated by its G-protein-coupled receptor, located on a variety of tissues including the brain, heart, kidney, and uterus (Loup et al., 1991; Gimpl and Fahrenholz, 2001). Polymorphisms of the gene encoding the OT receptor, *OXTR*, have been shown to contribute to individual differences in various social phenotypes (cf. Gimpl and Fahrenholz, 2001; Meyer-Lindenberg et al., 2011; Ebstein et al., 2012; Zink and Meyer-Lindenberg, 2012; Kumsta et al., 2013; Westberg and Walum, 2013). For example, *OXTR* single nucleotide polymorphisms (SNPs) have been associated with lower positive affect (Lucht et al., 2009), lower levels of responsiveness of mothers to their toddlers (Bakermans-Kranenburg and van IJzendoorn, 2008), lower empathy scores and increased stress reactivity (Rodrigues et al., 2009), non-verbal displays of prosociality (Kogan et al., 2011), and pair-bonding (Walum et al., 2012). *OXTR* SNPs have also been studied in relation with autism spectrum disorder (ASD; see Ebstein et al., 2012, for a review), with evidence that they contribute to risk for some phenotypes observed in ASD (Egawa et al., 2012; but see Tansey et al., 2010).

**Figure 2:**
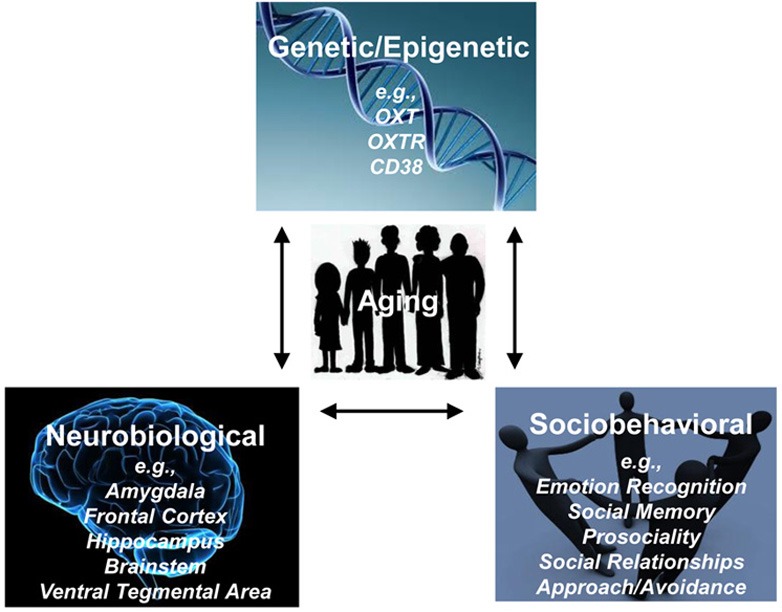
**Age-related Genetic, Neurobiological, Sociobehavioral Model of Oxytocin (AGeNeS-OT model)**.

Taken together, this review highlights the importance of simultaneously considering behavioral, neural, and genetic perspectives when examining OT's role in socioemotional functioning, as will be discussed in more detail below (see Figure 2). In addition, it raises five important caveats and informational gaps.

First, some of the effects associated with OT are inconsistent and come from small, homogeneous samples, creating a need for replication of key findings in larger, more representative samples.

Second, many of OT's effects seem to vary by individual difference variables such as the level of social proficiency (Bartz et al., 2011; but Guastella et al., 2013).

Third, there is increasing evidence suggesting that the effects of OT are dependent on context (Domes et al., 2007b) and influenced by early life experiences (see MacDonald, 2012, for a review). For example, women (Heim et al., 2008) and men (Meinlschmidt and Heim, 2007) who were abused or neglected as children showed altered OT system sensitivity as adults (e.g., decreased CSF level of OT; see also Winslow et al., 2003; Fries et al., 2005; but see Anderson, 2006; cf. MacDonald, 2012, for a review).

Fourth, due to both theoretical safety concerns using OT in women as well as the complexity introduced by OT's sex-specific effects, a large majority of studies conducted so far refer to men exclusively, even though there are growing indications that some of OT's effects may differ by sex (Savaskan et al., 2008; Guastella et al., 2009; Domes et al., 2010; Marsh et al., 2010; cf. MacDonald, 2012). This sex-specific pattern raises the possibility that the effects of OT on social cognition may be differentially regulated by gonadal steroids (estrogen and testosterone) or other sex-specific biological factors (Choleris et al., 2009; Gabor et al., 2012; see also Van Anders et al., 2011; see also Weisman and Feldman, 2013).

A fifth shortcoming in the current human literature on oxytocin—critical in the present context—is that current studies have almost exclusively been conducted with young adults. Given the aforementioned evidence of age-group differences in socioemotional functioning (Scheibe and Carstensen, 2010; Samanez-Larkin and Carstensen, 2011), a comprehensive examination of aging-related aspects of the OT system (including genetic, neurobiological, and behavioral aspects) is warranted (Huffmeijer et al., 2012).

## Oxytocin and aging

Despite a significant need for research addressing the growing older segment of the population, research on OT and aging is scarce and inconclusive. To date, the few studies that have addressed age-related differences in the OT system almost exclusively refer to non-human species with limited applicability to humans (Quinn, 2005). Also, studies conducted to date are characterized by large methodological differences in terms of species examined, OT parameters measured, brain regions targeted, etc., which makes a direct comparison difficult and a meta-analytic approach not feasible. Most importantly, a theoretical framework for generating hypotheses regarding age-related differences in the OT system (including changes in endogenous OT physiology, function, and differential response to exogenous OT) is entirely lacking (cf. Huffmeijer et al., 2012).

Table 1 provides a summary of the current studies on OT and aging. Whereas some studies suggest no noticeable effects of aging on the OT system (Fliers et al., 1985; Zbuzek et al., 1988; Wierda et al., 1991; Arletti et al., 1995), other studies report age-related change (Fliers and Swaab, 1983; Melis et al., 1992, 1995; Arsenijevic et al., 1995; Parker et al., 2010). Notably, some of the studies reporting comparability of the OT system across older and young subjects refer to peripheral OT levels (Fliers and Swaab, 1983; Zbuzek et al., 1988; Melis et al., 1992), whereas several of the studies documenting age-related change relate to central OT levels (Fliers and Swaab, 1983; Melis et al., 1992; Arsenijevic et al., 1995; Parker et al., 2010). Thus, it is possible that aging may change OT transmission in the CNS but not in the neurohypophyseal (peripheral) system (Melis et al., 1999). A summary of the evidence reported in Table 1 would be that current evidence does not allow yet a firm conclusion of the existence or direction of age-related changes in the OT system, leaving the question open to empirical examination.

**Table 1:** **Literature Review on Oxytocin and Aging**.

**Authors**	**Species**	**Age group**	**Measurement**	**Difference**	**Main findings**
**EVIDENCE OF STABILITY IN THE OT SYSTEM IN AGING**					
Arletti et al. (1995)	Rats (M)	O	Intraperitoneal OT injection	O = Y	Comparable improved social memory and anti-depressant effect of OT injection
Fliers et al. (1985)	Rats (M)	Y/O	OT fiber density	O = Y	Comparable OT fiber density in the brain
Wierda et al. (1991)	Human (M, F)	Y/O	Number of OT cells in PVN (post-mortem)	O = Y	Comparable numbers of OT-expressing cells in PVN (normal aging and Alzheimer's Disease)
Yu et al. (2006)	Rats (M)	Y/O	OT cell size and numbers in SON	O = Y	Comparable cell numbers, cell size, or reactive density of NOS-expressing neurons
**EVIDENCE OF CHANGE IN THE OT SYSTEM WITH AGE**					
Arsenijevic et al. (1995)	Rats (M)	Y/O	OT receptor binding	O < Y	Age-related decrease in binding to OT receptors in caudate putamen, olfactory tubercle, and ventromedial hypothalamic nucleus
Fliers and Swaab (1983)	Rats (M)	Y/MA/O	Plasma OT levels	O > Y (neurosecretory activity)	Age-related increase in OT secretion in PVN (but not SON); Comparable plasma OT levels
				O = Y (plasma levels)	
Keck et al. (2000)	Rats (M)	O	Intracerebral and peripheral OT release patterns	O > Y (peripheral)	Age-related increase in basal peripheral OT secretion and decrease in stress-induced intra-PVN OT secretion
				O < Y (intracerebral)	
Melis et al. (1992)	Rats (M)	Y/MA/O	OT levels	O < Y (CNS)	Age-related decrease in OT levels in septum and hippocampus; comparable OT levels in hypothalamus and hypophysis, and no change for plasma OT levels
				O = Y (HNS and plasma)	
Melis et al. (1995)	Rats (M)	Y/MA/O	OT-like immunoreactive peptides in thymic extract	O > Y	Age-related increase in content of OT-like immunoreactive peptides in thymic extract
Parker et al. (2010)	Rhesus monkeys (F)	Y/O	CSF OT levels	O > Y	CSF OT levels positively correlated with adult female age (but negatively correlated with infant age)
Zbuzek et al. (1988)	Rats (M)	O	Plasma and hypothalamic OT concentration	O = Y (plasma, hypothalamic concentration)	Comparable OT concentration in plasma and hypothalamus; age-related increase in secretory release of OT
				O > Y (secretory release)	

*Y, Young subjects; MA, Middle-aged subjects; O, Older subjects; M, Male; F, Female; OT, Oxytocin; PVN, Paraventricular nuclei of hypothalamus; SON, Supraoptic nuclei (SON) of hypothalamus; AVP, Arginine vasopressin; NOS, Nitric oxide synthase; CSF, Cerebrospinal fluid*.

To our knowledge, only one very recent study explicitly examined the effects of intranasal OT in a group of older men (mean age of 80 years) focusing on OT's effects on social engagement and physical health (Barraza et al., 2013). Results from this double-blind, placebo-controlled 10-day clinical trial suggested improvement in dispositional gratitude in older adults in the OT compared to the placebo group. In addition, the OT group had a slower decline in physical functioning and decreased self-reported fatigue than the placebo group. No changes in mood, cardiovascular states, or social activity and engagement patterns were observed across the study interval. Importantly, this study did not include a comparison group of young adults and did not extensively explore OT's effects on other aspects of socioemotional functioning. Thus, it is critical to follow up on these first promising findings regarding OT and aging and to conduct systematic examinations of age differences in baseline levels of OT. In addition, a comprehensive evaluation of both single-dose as well as longer-term administration of intranasal OT and its effect on socioemotional functioning in young and older men and women is warranted. Finally, these studies should take into account genetic variations related to OT.

## Oxytocin and socioemotional aging: age-related genetic, neurobiological, sociobehavioral model of oxytocin

**Figure 3:**
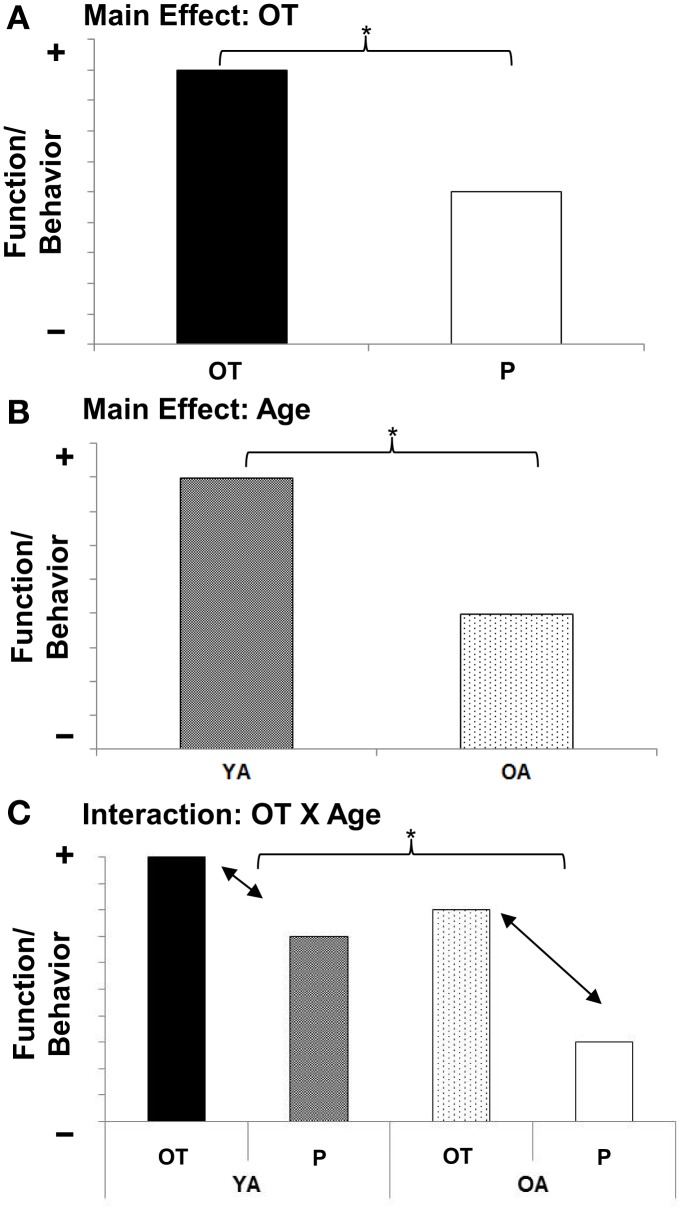
**(A)** Main effect for Age; **(B)** Main effect for Oxytocin; **(C)** Oxytocin X Age interaction effect. Schematic representation of guiding working hypotheses. YA, Young adults, OA, Older adults; OT, Oxytocin, P, Placebo.

Based on the following rationale, we propose an *OT X Age* interaction (see Figure 3C) as the guiding working hypothesis for future research on the role of OT in socioemotional aging: As mentioned above, there is early evidence that the beneficial effects of OT in socioemotional domains (see Figure 3A) vary by individual factors (Bartz et al., 2011). Notably, “preexisting social impairment” seems to play a role, in that more socially impaired individuals benefit more from OT than less socially impaired individuals (Bartz et al., 2010; Guastella et al., 2010; but see Bakermans-Kranenburg and van IJzendoorn, 2013). Also there may be a “ceiling effect,” a point beyond which OT cannot further improve social abilities (Bartz et al., 2011). As laid out above, older adults experience deficits in various socioemotional capacities (Scheibe and Carstensen, 2010; see Figure 3B), rendering them more socially impaired than young adults in some regards. Therefore, it may well be that OT is particularly beneficial in older compared to young adults (see Figure 3C).

However, an alternative hypothesis exists: As reported above, even though some aspects of socioemotional functioning (i.e., emotion recognition and memory for emotional information) decline with age, other aspects increase or remain stable. That is, given broad evidence for a positivity effect and for healthy socioemotional functioning in old age (Carstensen, 2006; Carstensen et al., 2011), as well as some evidence for increased trustworthiness in old age (Castle et al., 2012), on average, older adults can be described as highly positive, trustworthy, and prosocial. These characteristics may be adaptive in some contexts (e.g., social interactions within close relationships) but maladaptive in others (e.g., putting aging adults at greater susceptibility to fraud). This reasoning, combined with the current lack of proof that aging is associated with declines in the OT system and mixed evidence regarding OT's effect on cognitive performance (Heinrichs et al., 2004; Feifel et al., 2012), suggest the possibility that under certain circumstances OT may have harmful effects in older adults. Given that OT is currently being investigated in clinical populations such as schizophrenia (cf. MacDonald and Feifel, 2012, 2013), comprising samples of people who are late middle-aged, a thorough investigation of age-related aspects of the OT system-including beneficial or detrimental effects on outcome measures in socioemotional as well as cognitive domains-will be crucial.

As summarized above, the OT system is represented at genetic, neural, and behavioral levels (Meyer-Lindenberg et al., 2011). Furthermore, each of these levels and their functional interactions are influenced by the aging process. We therefore propose for future research in the domain of OT and socioemotional aging to adopt an *Age-Related Genetic, Neurobiological, Sociobehavioral Model of Oxytocin* (*AGeNeS-OT model*; Figure 2). In particular, this model suggests that a comprehensive examination of the central OT system should consider interactions between OT-related genes (*OXT, OXTR, CD38;* Meyer-Lindenberg et al., 2011; Sauer et al., 2013), the brain (e.g., amygdala, frontal cortex, brainstem, ventral tegmental area; Pedersen et al., 1994; Kirsch et al., 2005; Balleine et al., 2007; Baumgartner et al., 2008; Gamer et al., 2010), and behavior (e.g., social memory, emotion identification, approach/avoidance biases; Rimmele et al., 2009; Domes et al., 2010; Lischke et al., 2011), by combining genetics, functional and structural brain imaging, and sociobehavioral measures. Crucially, the model proposes that interactions between neuroendocrine and sociobehavioral factors need to be considered from a developmental perspective, taking age variations into account. Along these lines, the model offers a theoretical framework to address vital research questions: (1) Are OT-related genotypes associated with composition and quality of social networks in the elderly? How do brain structures involved in social processing such as mPFC and OFC, temporoparietal junction, or amygdala mediate these relationships? (2) Is older adults' increased social avoidance compared to approach motivation represented in neural processing differences in brain networks involving PFC and amygdala? To what extent do these associations interact with OT-related genotypes? (3) Are detrimental effects that early abuse has on morbidity and mortality in the elderly moderated by OT-related genotypes or OT levels? How is this relationship structurally and functionally represented in the brain? (4) Are effects of social relationships on cognitive functioning in the elderly mediated by the OT system (either OT-levels or OT-related genotypes)? Do structural changes in brain regions such as the hippocampus underlie this relationship?

In the attempt to provide a concrete empirical application of the *AGeNeS-OT Model*, we here present a preliminary report of an experiment in which we examined associations between *OXTR* polymorphisms, brain activity and behavioral response during reading of facial emotions in young and older adults. This exploratory, secondary data analysis was based on our group's previous finding of increased activation in ventromedial PFC (vmPFC) during emotion identification of happy compared to angry faces and increased dorsomedial PFC (dmPFC) activity to angry compared to happy faces (Ebner et al., 2012; see also Keightley et al., 2007) in both young and older adults. In the present set of analyses, we examined the extent to which these processing differences in mPFC would be further qualified when considering *OXTR* polymorphisms in both of the age groups. In particular, we examined (1) the extent to which *OXTR* polymorphisms were associated with differences in young and older adults' brain activity in bilateral mPFC (Haxby et al., 2000, 2002; Pessoa and Adolphs, 2010; Ebner et al., 2012) during a facial emotion reading task; and (2) the extent to which *OXTR* polymorphisms were associated with young and older adults' ability to read facial emotions.

Young [*n* = 25, 12 females, *M* = 25.1 years (*SD* = 3.6, range = 20–31)] and older [*n* = 29, 17 females, *M* = 68.3 years (*SD* = 2.8, range = 65–74)] healthy participants underwent fMRI on a 3T Siemens Magnetom TrioTim scanner, while identifying happy, neutral, and angry facial emotions (see Ebner et al., 2012, for details on participants, study design, and image acquisition). Participants were subsequently genotyped by KBioscience (http://www.kbioscience.co.uk) using KASPar methodology for 14 *OXTR* single nucleotide polymorphisms (SNPs in order from the 3′ to the 5′ end: rs7632287, rs6770632, rs1042778, rs237887, rs2268493, rs2254298, rs53576, rs237897, rs4686302, rs4564970, rs2301261, rs2268498, rs2270465, rs75775), previously shown to be associated with social behavior (Apicella et al., 2010; Meyer-Lindenberg et al., 2011; Ebstein et al., 2012; Walum et al., 2012; Westberg and Walum, 2013).

**Figure 4:**
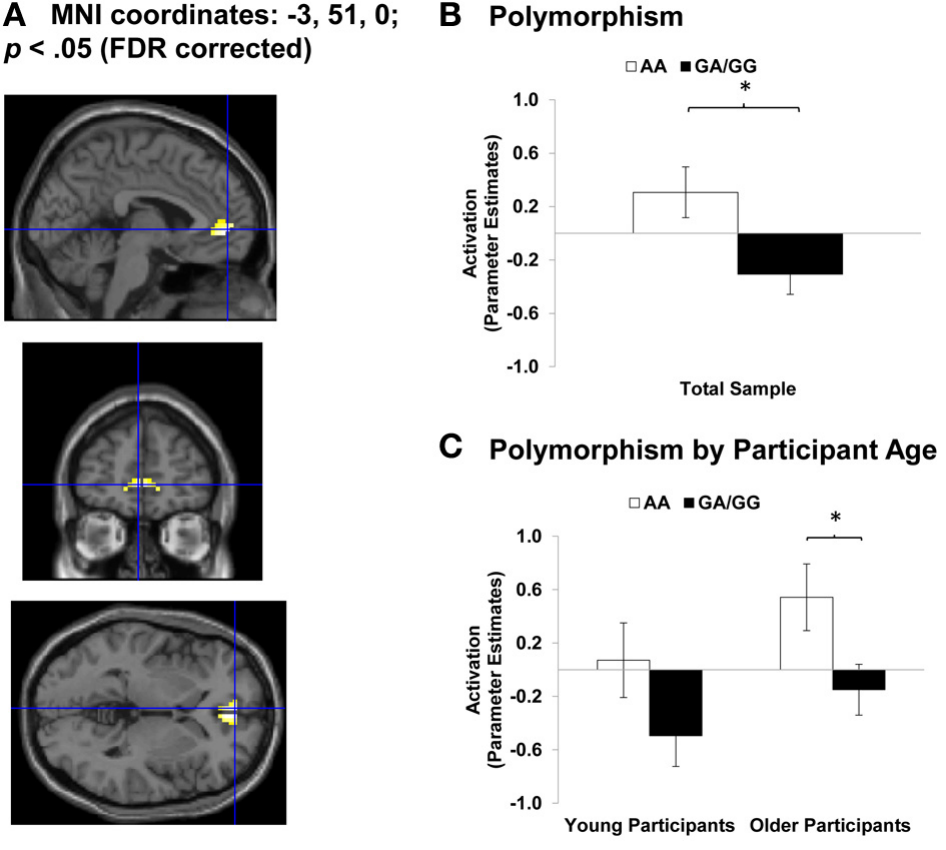
**Area of Anterior Cingulate Cortex (ACC) showing Happy Faces > Angry Faces (T-contrast). (A)** Left ACC (BA 32, 10; MNI: *x* = −3, *y* = 51, *z* = 0; cluster size: 26 voxels; maximum *T*-value for cluster: 4.19). The region of activation represents the T-map of the contrast; it is displayed on the standard reference brain in SPM. The crosshair indicates the peak voxel (local maximum) within the region of activation. **(B)** Bar graphs show the mean left ACC parameter estimates (beta values) separately for *OXTR* rs237887 AA and GA/GG carriers. **(C)** Bar graphs show the mean left ACC parameter estimates (beta values) separately for *OXTR* rs237887 AA and GA/GG carriers and young and older participants, respectively; betas depicted were extracted for each individual from a 5-mm sphere around the local maximum within the region of activation and averaged to produce a single value for each condition of interest, respectively. Note. ^*^p < 0.05. Error bars represent standard errors of the between-group differences.

Data from this event-related fMRI study was analyzed using Statistical Parametric Mapping (SPM5; Wellcome Department of Imaging Neuroscience) and pre-processing and data analysis was conducted as reported in Ebner et al. (2012). The following *T-contrasts* were specified across young and older adults, based on our previous findings (Ebner et al., 2012): (1) *Happy Faces > Angry Faces*, (2) *Angry Faces > Happy Faces*. We focused on select regions of interest (ROIs: bilateral medial frontal gyrus and anterior cingulate gyrus) in which we had previously seen processing differences for happy vs. angry faces, at a threshold of *p* < 0.05, FDR corrected. For each region of activation identified by these two contrasts, peak voxel beta values were extracted for each participant to produce a single value for each condition of interest. These values are depicted in the bar graphs of Figure 4. In the fashion of follow-up *F*- and *t*-tests (*p* < 0.05), for each of the 14 *OXTR* SNPs that were genotyped, we examined differences in brain activation between polymorphisms across the total sample as well as separately for young and older adults. The most consistent associations found in these analyses were in relation to *OXTR* rs237887 (cf. Lerer et al., 2008; Israel et al., 2009; Liu et al., 2010; Lori et al., 2012; but see Apicella et al., 2010).

*OXTR* rs237887 AA carriers (*n* = 10 young participants; *n* = 10 older participants) and GA/GG carriers (*n* = 15 young participants; *n* = 19 older participants) were comparable in terms of chronological age, level of education, cognitive status (e.g., Mini Mental State Examination; Folstein et al., 1975; 2-Back Digits Task; Kirchner, 1958; Verbal Fluency Task; Lezak, 1995), and affective variables (Geriatric Depression Scale; Brink et al., 1982; Gottfries, 1997; State-Trait Anxiety Inventory; Spielberger et al., 1970).

For the contrast *Happy Faces > Angry Faces*, we found greater BOLD response to happy compared to angry faces in bilateral anterior cingulate cortex (ACC; MNI: *x* = 3, *y* = 45, *z* = 0 and *x* = −3, *y* = 51, *z* = 0) and bilateral mPFC (MNI: *x* = 3, *y* = 60, *z* = −3 and *x* = −3, *y* = 57, *z* = −3). Figure 4A shows brain activity in left ACC (MNI: *x* = −3, *y* = 51, *z* = 0) for this contrast. To then examine associations between *OXTR* rs237887 polymorphisms and brain activity during facial emotion identification of happy vs. angry faces in young and older adults, we conducted follow-up univariate ANOVA collapsed across young and older participants on extracted beta values at the peak voxel of activation. Left ACC activity was greater for AA carriers than GA/GG carriers [*F*_(1, 51)_ = 6.51, *p* = 0.014, η^2^_*p*_ = 0.11; see Figure 4B]. More interestingly, however, this effect was more pronounced in older than young adults, as tested in univariate ANOVAs conducted separately within young and older participants [Young participants: *F*_(1, 23)_ = 2.38, *p* = 0.136, η^2^_*p*_ = 0.09; Older participants: *F*_(1, 26)_ = 3.09, *p* = 0.035, η^2^_*p*_ = 0.16; see Figure 4C]. A comparable pattern of results was found for right ACC [*F*_(1, 51)_ = 6.34, *p* = 0.015, η^2^_*p*_ = 0.11]. In addition, the results for left [*F*_(1, 51)_ = 3.24, *p* = 0.078, η^2^_*p*_ = 0.06] and right [*F*_(1, 51)_ = 1.29, *p* = 0.261, η^2^_*p*_ = 0.03] mPFC pointed in the same direction but were not significant.

ACC is a brain region associated with affective processing (Bush et al., 2000; Amodio and Frith, 2006; Ebner et al., 2012), suggesting that AA compared to GA/GG carriers may process happy compared to angry faces more affectively. This interpretation was further supported by the finding that AA-genotype carriers of *OXTR* rs237887 (*M* = 1111 ms, *SD* = 171 ms) were faster at labeling happy expressions than individuals carrying a G-allele [*M* = 1212 ms, *SD* = 173 ms; *F*_(1, 50)_ = 4.26, *p* = 0.044, η^2^_*p*_ = 0.08], with comparable effects in young and older participants. No comparable effect was found for accuracy in emotion expression identification. However, interestingly, greater recruitment of right ACC in individuals carrying a G-allele was positively correlated (*r* = 0.35; *p* = 0.049) with accuracy in reading happy faces but uncorrelated in AA-genotype carriers (*r* = 0.05; *p* = 0.838). This suggests that GA/GG carriers, as the group who needed more time on the task, benefitted from recruiting ACC during the facial emotion reading task. This positive brain-behavior correlation in GA/GG carriers was comparable in young and older participants (Fisher's *z* = −0.42; *p* = 0.337).

For the contrast *Angry Faces > Happy Faces*, we found greater BOLD response to angry compared to happy faces in left mPFC (MNI: *x* = −6, *y* = 15, *z* = 51). In a follow-up univariate ANOVA collapsed across young and older participants on extracted beta values at the peak voxel of activation, activity in left mPFC did not vary by *OXTR* rs237887 polymorphism (*p* > 0.05).

To our knowledge this is the first study that considers young and older participants in a genetic-neuro-behavioral examination of facial emotion processing, as suggested in the *AGeNeS-OT model*. Though this secondary data analysis was largely exploratory and replication in a larger independent sample of young and older adults is warranted, our study provides some first indication of a role of *OXTR* rs237887 in reading positive compared to negative facial expressions, with some variation as a function of the age of the participant. Intriguingly, *OXTR* rs237887 has previously been associated with susceptibility for ASD (Liu et al., 2010), prosocial behavior (Israel et al., 2009, but see Apicella et al., 2010), and face recognition (Lori et al., 2012). We found improved processing of happy compared to angry faces for AA carriers compared to GA/GG carriers, as reflected in their faster response time in reading happy faces and their increased recruitment of ACC during emotion reading of happy compared to angry faces. Examining young and older participants separately, this increased activation of ACC in AA compared to GA/GG carriers was more pronounced in older than young participants. This is very interesting given broad evidence of preferential processing of positive over negative information in older compared to young adults (Mather and Carstensen, 2005). In addition, our findings suggest that GA/GG carriers' ability to correctly identify happy faces improved when recruiting ACC during the task.

## Future trends in research on oxytocin and socioemotional aging

Taken together, this research review indicates that a targeted investigation of age-related changes in the OT system—especially one that considers genetic, neural, and behavioral processes—has the potential to substantively increase our understanding of socioemotional change in aging. We believe that our *AGeNeS-OT model* will be a fruitful conceptual basis in that it raises a set of vital research questions necessary to refine our understanding of OT-related dynamics in aging in socioemotional contexts (see Box 1). In addition, future research along those lines has great potential to inform both pharmacological and psychosocial interventions targeting social and emotional dysfunction in the elderly. In particular, there is an increasing body of research suggesting a significant role of OT in the context of various disorders characterized by socioemotional dysfunction such as social-bonding deficits or related to social anxiety and stress (Zetzsche et al., 1996; Heinrichs et al., 2003; Taylor et al., 2006; see MacDonald and Feifel, 2012, for an overview), deficits with great relevance in an aging context. Thus, future research toward implementation of pharmacological neuropeptide treatments with the potential to decrease emotional and social stress, anxiety, and depression (Arletti and Bertolini, 1987; Carter and Altemus, 1997) will be important. These interventions may consequently promote positive social interaction and willingness to engage in more frequently rewarding social risks (Heinrichs et al., 2003; Kosfeld et al., 2005), improving health and life quality up until late in life.

Box 1 **Questions for future research.**

1. Is aging accompanied by increases or decreases in central and peripheral release of OT?
2. Does the dynamic activity of the OT system change with age and, if so, how and why?
3. Do age-related differences in OT system dynamics underlie age-related differences in socioemotional functioning? If so, how do these changes frame our understanding of the age-associated changes in important social skills (i.e., reading facial emotions, face memory, approach, and avoidance behavior)?
4. How do OT-related individual genetic (and epigenetic) differences interact with neural and behavior age-related changes in socioemotional domains?
5. Does the OT system mediate some of the effects of adverse early experience on health and well-being? How does this play out in old age?
6. Does the OT system mediate some of the salutary psychological and health effects of ongoing social relationships (both intimate and larger social networks)? To what extent do age-related changes in social relationships influence these effects?
7. How do sex differences in OT system dynamics play out in the context of aging? For example, what is the role of age-related changes in estrogen and testosterone?
8. Does the OT system have a role in age-related changes in cognition and memory?
9. Might OT be an effective treatment for conditions like social anxiety or depression in the elderly? Would such treatment improve quality of life?
10. Might older adults be at increased risk of OT-related side effects (i.e., hyponatremia) with chronic dosing?

### Conflict of interest statement

The authors declare that the research was conducted in the absence of any commercial or financial relationships that could be construed as a potential conflict of interest.
